# Smoke Obscuration Measurements in Reduced-Scale Fire Modelling Based on Froude Number Similarity

**DOI:** 10.3390/s19163628

**Published:** 2019-08-20

**Authors:** Wojciech Węgrzyński, Piotr Antosiewicz, Tomasz Burdzy, Mateusz Zimny, Adam Krasuski

**Affiliations:** 1 Fire Research Department, Building Research Institute (ITB), Filtrowa 1 St., 00-611 Warsaw, Poland; 2 Faculty of Mining and Geoengineering, AGH University of Science and Technology, Al. Mickiewicza 30, 30-059 Kraków, Poland; 3 Faculty of Fire Safety Engineering, The Main School of Fire Service (SGSP), Słowackiego 52/54 St. Warsaw, 01-629 Warsaw, Poland

**Keywords:** smoke, fire, smoke obscuration, visibility in smoke, Froude number, scale modelling

## Abstract

A common method for investigating various fire- and smoke-related phenoma is a reduced-scale fire modelling that uses the conservation concept of Froude number as its primary similarity criterion. Smoke obscuration measurements were not commonly used in this approach. In this paper, we propose a new type of optical densitometer that allows for smoke obscuration density measurements on a reduced-scale. This device uses a set of mirrors to increase the optical path length, so that the device may follow the geometrical scale of the model, but that still measures smoke obscuration as if it were in full scale. The principle of operation is based on the Bougher-Lambert-Beer law, with modifications related to the Froude number-based scaling principles, to streamline the measurements. The proposed low-budget (< $1000) device was built, calibrated with a set of the reference optical filters, and used in a series of full- (1:1) and reduced-scale (1:4) experiments with n-Heptane fires in a small compartment. The main limitation of this study is the assumption that there is similar soot production in full- and reduced-scale fires, which may not be true for many Froude-number scaling applications. Therefore, it must be investigated in a case-by-case basis. In our case, the results are promising. The measured obscuration in the reduced-scale had a 10% error versus averaged measurements in full-scale measurements. Moreover, there were well represented transient changes of the smoke layer optical density during the combustion and after the smoke layer settled.

## 1. Introduction

Fire-building is a complex phenomenon studied by multiple research groups around the world. Providing fire safety to buildings and their users is often the driving force behind many design choices by various stakeholders in the development of civil engineering structures [1]. The most valuable contributions to our understanding of fire phenomena in buildings that come from full-scale fire experiments, prime examples of which are [2,3,4,5]. However, such experiments are associated with high costs, lengthy preparation, and inevitable uncertainty due to the inability to repeat the test under the same conditions. Due to abovementioned causes (albeit mainly due to high costs), fire scientists must resort to other popular methods to investigate fire phenomena—primarily numerical modelling with the use of Computational Fluid Dynamics (CFD) method [6] and reduced-scale fire experiments based on the Froude number similarity criterion [7]. The instrumentation of the latter is the scope of this research paper.

Smoke (The term “smoke” in this paper is defined as any mixture of soot, other combustion products, and surrounding air, as a result of turbulent mixing that happens in a buoyant plume above the fire.) obscuration is among the universal measures in any building related fire experiment, along with temperature, surface temperatures, net heat fluxes, and concentration measurements. Following the theory of Jin [8,9], the smoke obscuration may be reasonably easily transformed into visibility in smoke, which is among the most widely used tenability criterions for the design of fire safety features in buildings [10]. It can be argued that in most engineering projects the smoke visibility is the first parameter to reach its tenability limit, thus becoming the limiting factor for the building design.

**Figure 1:**
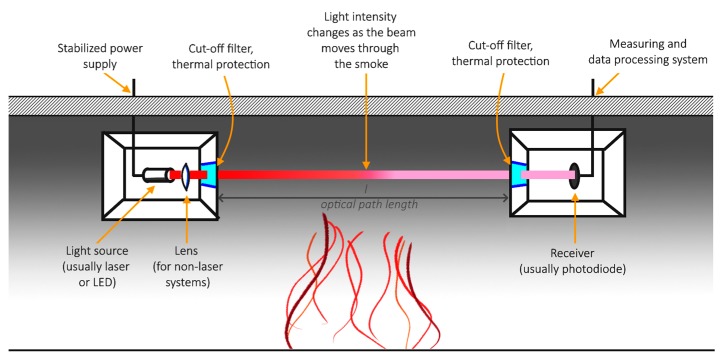
The idea of optical densitometers for smoke obscuration in fire experiments.

In case of fire experiments, the smoke obscuration is measured as the ratio of the intensity of light received when passing through the smoke to the initial intensity, also known as the transmittance of the medium. The relation between the mass density of smoke and the transmittance over a distance can be calculated with the Bougher-Lambert-Beer law Equation (1), by comparing the current on the photodiode in ambient and experiment conditions. The illustration of this concept is visualised in Figure 1.

(1) $$\frac{I}{I_{0}}=e^{-\sigma_{s}ml}$$

It is recognised that this approach has many limitations [10], among which are as follows: (a) it should be used for monochromatic light; (b) it should be used for single or parallel light sources; and (c) the light absorption is considered dominant over light scattering effects. Nevertheless, this approach is widely used in fire experiments due to fairly easy instrumentation of the optical densitometers. An example of such a device built ad hoc for a fire experiment is given in [3].

Although commonly used in full-scale experiments and numerical simulations, measurements of smoke obscuration effects are not performed in reduced-scale models. To the best of the authors’ knowledge, we were not able to identify any study in which such measurements were pursued. This may be attributed to the fact, that in reduced-scale models, the amount of smoke within the compartment is significantly smaller than in their full-scale counterpart, thus leading to smaller smoke obscuration value (if measured at the same distance as in full scale). Based on the practical experience of the authors, the smoke obscuration measurements at very low smoke densities and short distances (scaled to the reduced geometrical scale) require optical densitometers with high sensitivity that come at a high cost. This is often problematic, as the optical densitometer in fire environment is prone to damage by hot gasses and smoke, and thus low-budget devices that are easy to replace are preferred. The effect of smaller mass density could be potentially mitigated with the use of densitometers with the long optical path. However, such a device will not follow the geometrical-scale of the reduced-scale model and thus will result in measurements of smoke obscuration averaged over a longer distance than in the full-scale model.

Furthermore, if one wants to model the visibility in the small-scale, problems with the scaling of the fire to preserve the soot yield in the reduced-scale may occur. The size of the fire may affect combustion efficiency, which will strongly affect the soot production and particle distribution. The researchers should seek experimental proof regarding soot yield generation of their model fire in various geometrical sizes (an example can be found in [11,12]) to reduce the uncertainty. Another solution is to replace the source of fire with a source of clean hot air / helium with a known amount of aerosol injected and scaled based on the Froude number modelling. Moreover, it should be noted that after precise calibration the apparatus presented in this research may also be used to determine the soot yield of a scaled-down fire if all other requirements for Froude number scaling (Section 2) are preserved and the specific smoke mass density for the chosen wavelength is known. In such a case, the optical densitometer will act similar to the device used in the Tewarson apparatus used to determine soot yield of fires with different ventilation factors [13].

This paper introduces a new concept of low-budget optical densitometer that can measure smoke obscuration in reduced-scale models while conforming to the geometrical scale of the model study. The device uses an array of mirrors to increase the length of the optical path, so that the optical path in the reduced-scale is equal to the path length in full scale.

## 2. Froude Number Based Fire Modelling

Dimensionless scaling techniques are in everyday use in fire safety science since the mid-XXth Century. Studies by Thomas et al. [14] or Prahl and Emmons [15] are considered as fundamental for the development of modern fire science and the whole area of smoke control. The concept of reduced-scale modelling was summarized by Quintiere in [7] and more recently in [16], while the 29 dimensionless groups that are the foundation of scale fire models were derived by Williams (1969) and published in [17]. It is worth noting that in the original work by Williams, the dimensionless smoke obscuration was not derived, although the original 29 dimensionless groups include the conservation of mass and chemical species. It should also be noted that here the authors focus on the scaling of smoke layer formation in an enclosed compartment during relatively short experiments. Thus, many of the dimensionless groups (primarily related to heat transfer in solids, pyrolysis, and chemical reaction kinetics) are omitted on purpose. For a more thorough introduction to Froude number-based reduced-scale fire modelling, the reader is kindly referred to [16]. The reduced-scale fire modelling of smoke plumes and smoke layers may be achieved primarily with buoyant hot air plumes over a source of fire, but also with less popular techniques: water-kerosene mixtures, water-saltwater mixtures, helium plumes in air, and air plumes in sulfur hexafluoride (SF_6_). In this paper, only the hot air buoyant plumes over reduced-scale fire sources are considered.

Froude number (Fr) is a dimensionless number defined as the ratio of the forces of inertia to the external field (in case of fire: buoyant forces):

(2) $$\mathrm{Fr}=\frac{u_{0}}{\sqrt{g_{0}l}}$$

Two fires can be considered similar if the following requirements are met:

- Froude number of both of the fires is equal;
- all geometrical features related to the fires are scaled with the same scale;
- the fire is occurring at well-ventilated conditions, i.e., the combustion is not significantly influenced by the reduced-scale, and the combustion efficiency in full and reduced-scale is similar;
- the flow in the buoyant plume is turbulent, and the Reynolds (Re) number for flow in the reduced-scale model is Re > 10,000.

The abovementioned requirement related to the Reynolds (Re, Equation (3)) number is introduced, as the conservation of Froude and Reynolds numbers in the same model may be difficult.

(3) Re=u0lρμ

It can be noted that scaling the Reynolds number while following Froude relationship would require scaling of the kinematic viscosity of the medium and thus rendering the method impractical. However, if the flow is highly turbulent, the Re number will have a limited effect on the fluid dynamics of the smoke plume or layer. Thus, its omission can be considered justified.

The geometrical scale of the model may be defined as the ratio of the characteristic dimensions of the scaled-down model (index “*m*”) to the corresponding dimensions of the full scale (index “*f*”) Equation (4).

(4) $$\frac{x_{m}}{x_{f}}$$

**Figure 2:**
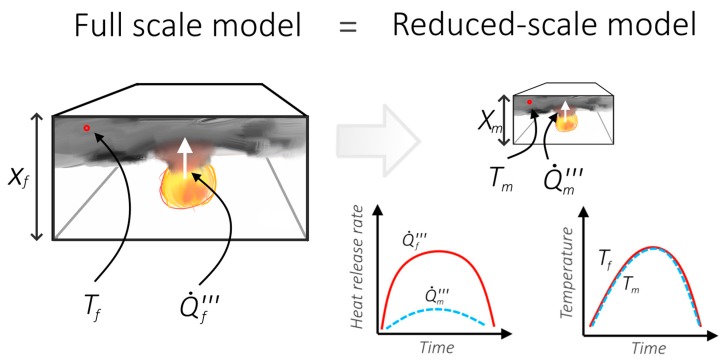
The idea of the Froude number reduced-scale fire modelling. Illustrating the concept of scaling down the heat release rate of the fire to model a fire, that causes similar consequences in the scaled-down (geometrical) compartment.

If the Froude similarity criterion is met and Reynolds criterion is satisfied, other relevant parameters that describe the flow of mass and heat in the compartment will scale as below. The illustration of scaling principles is shown on Figure 2.

1. The heat release rate of the fire [kW]
  (5) $$\frac{{\dot{Q}}_{m}}{{\dot{Q}}_{f}}={\left(\frac{x_{m}}{x_{f}}\right)}^{5/2}$$
2. Time [s]
  (6) $$\frac{t_{m}}{t_{f}}={\left(\frac{x_{m}}{x_{f}}\right)}^{1/2}$$
3. Energy [kJ]
  (7) $$\frac{E_{m}}{E_{f}}={\left(\frac{x_{m}}{x_{f}}\right)}^{3}$$
4. Air velocity [m/s]
  (8) $$\frac{V_{m}}{V_{f}}={\left(\frac{x_{m}}{x_{f}}\right)}^{1/2}$$
5. Mass flow [kg/s]
  (9) $$\frac{{\dot{m}}_{m}}{m_{f}}={\left(\frac{x_{m}}{x_{f}}\right)}^{3}$$
6. Temperature [K]
  (10) $$T_{m}=T_{f}$$

The production of smoke in the reduced-scale source will be the mass loss at a reduced scale Equation (9) multiplied by relevant yield factor. Here, we assumed the same combustion efficiency between full- and reduced-scale models. In the case of soot production in the combustion of n-Heptane, used as a fuel in the experimental section of the paper, the soot yield factor of a well-ventilated fire amounts to Y_soot_ = 0.037 [g/g]. This assumption may require additional verification if the device is used for larger fires or small scales. The smoke production follows the relation of $\sim {\left(\frac{x_{m}}{x_{f}}\right)}^{3}$, similar to the volume of the model. If we investigate smoke production in the full-scale model domain, we can write that the change of mass density in the volume and time follows relation Equation (11).

(11) $$\frac{dm}{dt}=\frac{1}{V}\int_{t_{0}}^{t}\frac{\dot{Q}}{\Delta H_{c,eff}}Y_{soot}dt$$

Following dimensional analysis of variables of this relation, we find that the scaling the smoke production, per volume of the compartment and the total time of the release, the scaling coefficients Equation (12) equal to ${\left(\frac{x_{m}}{x_{f}}\right)}^{\frac{5}{2}}{\left(\frac{x_{m}}{x_{f}}\right)}^{\frac{1}{2}}{\left(\frac{x_{m}}{x_{f}}\right)}^{-3}$, which amounts to 1.

(12) $$m_{m}\sim Q{\left(\frac{x_{m}}{x_{f}}\right)}^{\frac{5}{2}}t{\left(\frac{x_{m}}{x_{f}}\right)}^{\frac{1}{2}}\frac{1}{v{\left(\frac{x_{m}}{x_{f}}\right)}^{3}}$$

This means that mass density of the pollutant (after release in the same dimensionless time, in the scaled-down volume) will follow a similar relation, like the temperature, and it can be written that Equation (13):

(13) $$\frac{m_{m}}{m_{f}}=1$$

## 3. Measuring Smoke Obscuration in Reduced-Scale Model

Measurements of the optical density in fluids are common in the world of science and the first densitometers based on the beam comparison and collimation [18] or photo-electric cells [19] precede the invention of photodiodes used in modern densitometers. Similar devices were widely used in fire science for measuring the smoke obscuration effects, especially after the quantification of smoke visibility effects by Jin [8,20]. A further breakthrough came with the quantification of the specific mass extinction coefficient for a wide array of fuels [21]. Advanced optical densitometers operating at a broad wavelength spectrum can also be used for optical measurements of soot volume fractions, based on different absorption of light by different size soot particles [22]. The same principle is also used to measure soot propensity in combustion processes [23]. An interesting approach in optical densitometry is to use infrared radiation generated by a background signal array to measure smoke transmittance of smoke clouds in field experiments. Such an approach was described in [24].

A critical parameter for the estimation of transmittance is the specific mass extinction coefficient of smoke. It is known that the different sizes of soot particles can absorb different light waves and thus the specific mass extinction coefficient in the function of the length of the light wave [25,26]. However, for the practical purpose of measurements, and to maintain the similarity to most widely used CFD model used in fire science (FDS [27]), the specific mass extinction coefficient value of $\sigma_{s}=8.71$ m²/g is used here, as determined by Mulholland and Croarkin [21].

In the following Equation (14), the mass density of smoke in the reduced-scale model will be similar to the local mass density of smoke in the full model and can be calculated using transmittance measured:

(14) $$m_{f}=\frac{-\ln\left(\frac{I}{I_{0}}\right)}{\sigma_{s}l}$$

Assuming that the size of the reduced-scale densitometer follows relation Equation (4), the dimensions of the densitometer will be:

(15) $$l_{m}=l_{f}\left(\frac{x_{m}}{x_{f}}\right)$$

To achieve the same optical path length as in full scale, but within this reduced geometrical dimensions, *n* passes of light through the medium are required Equation (16).

(16) $$n={\left(\frac{x_{m}}{x_{f}}\right)}^{-1}$$

**Figure 3:**
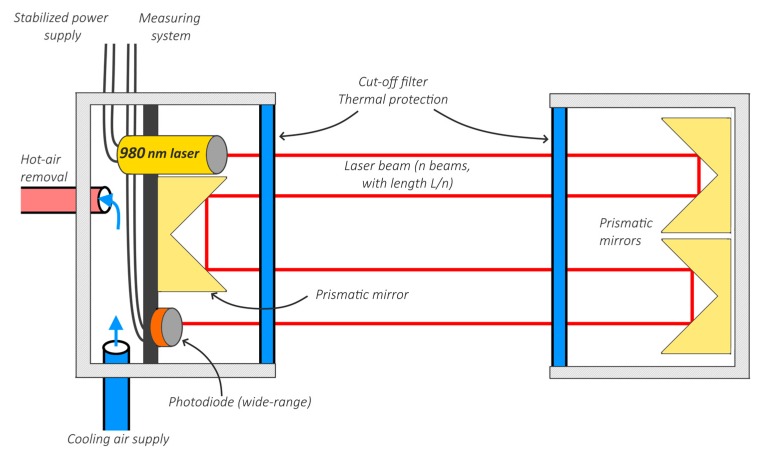
Dividing the optical path with a length of *l* into *n* beams with a length of *l/n*, to measure the transmittance in the reduced-scale model as in the full scale, based on Equations (14) and (16).

If this is met, the same transmittance should be measured in reduced-scale and the full-scale model. In further considerations, *n* is the number of sub-divisions of the optical path length into smaller segments (each with the length *l/n*). This idea is illustrated in Figure 3.

In practical use, the total transmittance measured at the receiver sensor will also be influenced by:

- The transmittance of the optical filters shielding the optical elements from smoke (*τ_f_*), which can also be used to filter out other wavelengths than one used for measurements;
- reflectance (*R_m_*) of the mirrors or prisms used in the device.

The total transmittance measured by the sensor will be:

(17) $$\frac{I}{I_{0}}=e^{-\sigma_{s}m_{s}l}{\left(\tau_{f}\right)}^{2n}{\left(R_{m}\right)}^{\left(2n-2\right)}$$

However, in practical measurements the term ${\left(\tau_{f}\right)}^{2n}{\left(R_{m}\right)}^{\left(2n-2\right)}$ can be considered as a constant value, describing the obscuration caused by the optical system of the sensor. If the initial value of the current (*I*_0_) is measured before the experiment on a completely assembled sensor, and thermal stability of the laser/photodiode is maintained during the experiment, the measured obscuration density will depend only on the mass smoke density.

## 4. Prototype Testing

### 4.1. Construction of the Prototype Device

A prototype device of optical densitometer for reduced-scale fire research was built. Stock plastic casings (IP class 65) were used to shield the electronic components from the smoke. The components of the system were:

- Laser diode module type LW-980-50-C12-DI;
- Photodiode OSRAM type IR PIN; TO5; 850 nm; 400–1100 nm; 55°; THT; 2n A;
- Three pieces of prismatic mirror type HRS 1015-AG—1” × 1” Hollow roof prism mirror, Ultrafast-Enhanced Silver;
- Two pieces of optical cut-out filter 800 nm, 50.0 × 50.0 mm sq., unmounted, Long Wave Pass Edge Filter.

Additionally, a photodiode amplifier system was built using the following elements:

- Modular switches;
- Modular symmetric power supply unit (± 15 V) and a power supply unit 15 W, 12 V, and 1.25 A;
- Symmetric transformer 2 × 18 V;
- Microcircuit LM 725;
- Potentiometer;
- Resistors (100 Ω–100 kΩ);
- Ceramic capacitors (100, 220 and 500 pF);
- An electrolytic capacitor (4.7 µF).

The electrical schematic of the amplifier may be shared upon request. The data was recorded with GL840 Graphtec logger (optical densitometers) and PicoLog data logger (thermocouples).

**Figure 4:**
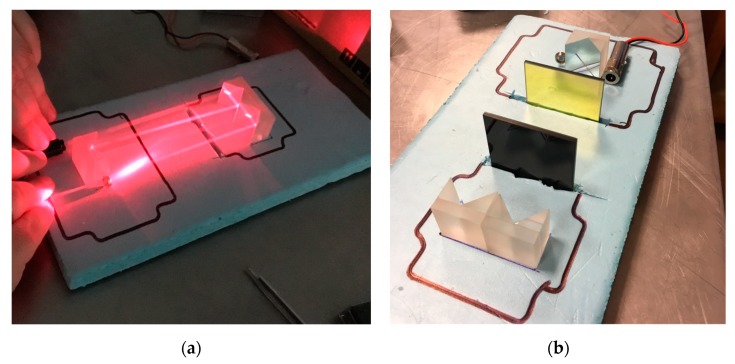
A prototype of the optical densitometer for reduced-scale fire research before the final assembly: (**a**) Illustration of the idea of the operation of the densitometer (with a 633 nm laser); (**b**) pre-assembly fitting of important optical components of the device.

A mockup illustration showing the operating idea and the simplified setup of the critical optical elements of the prototype is shown in Figure 4.

**Figure 5:**
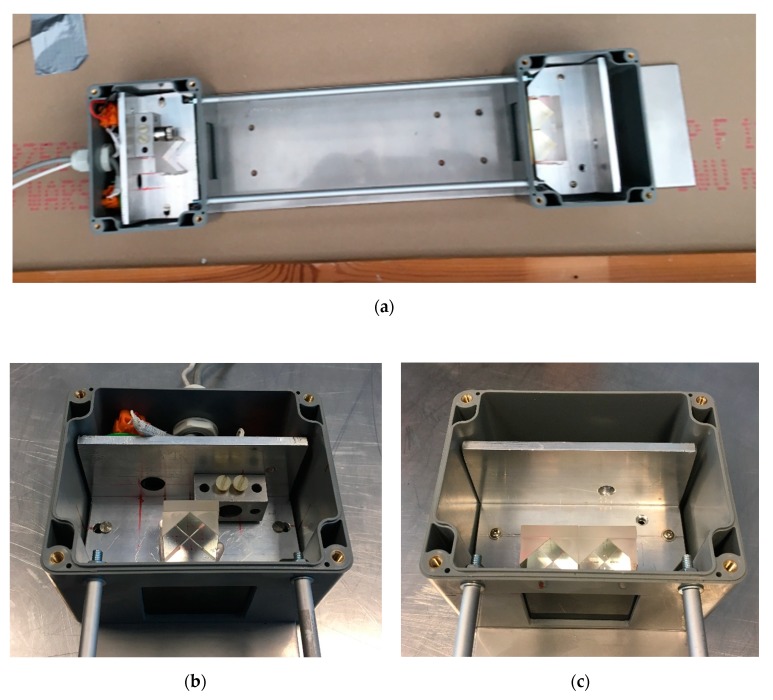
The final prototype of the optical densitometer for reduced-scale fire experiments. (**a**) Overview of the device; (**b**) close up of the laser-photodiode component; and (**c**) close up of the reflecting component.

After pre-testing, the prototype was mounted inside of smoke-proof casings, with cooling system for its electrical components and an additional support aluminium frame to assure the rigid behaviour of the structure. Illustration of the final prototype is shown in Figure 5.

### 4.2. Calibration of the Prototype Device

**Figure 6:**
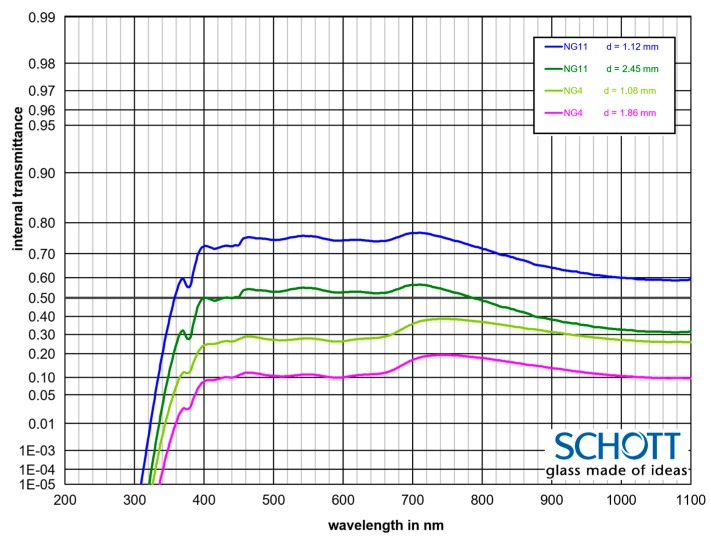
Internal transmittance of the calibration filters, calculated with a tool provided by the manufacturer (https://www.schott.com/advanced_optics/).

The calibration of the prototype device was performed using a set of four SCHOTT^®^ optical filters with a known transmittance, Figure 6. In the calibration procedure, each of the filters was manually placed to block respectively 1, 2, 3, or 4 paths of the light emitted by the laser. This measurement was repeated three times for each measurement point to limit the error of the process. To assess if the spatial location of the calibration filter affects the results, the calibration procedure was repeated for placement of the filter in front of the laser, inside of the beam between laser and reflecting units, and directly in front of the photodiode. Subsequent repetitions of this procedure led to similar results, confirming that the spatial location of the filter does not have a significant effect on the measurements.

The transmittance of the calibration filters and reflectance of the prism at 980 nm, as defined in their technical specifications were as follows:

- NG11 × 1.12     τ = 0.606
- NG11 × 2.45     τ = 0.334
- NG4 × 1.08       τ = 0.276
- NG4 × 1.86       τ = 0.109
- Prismatic mirror   R = 0.985.

**Figure 7:**
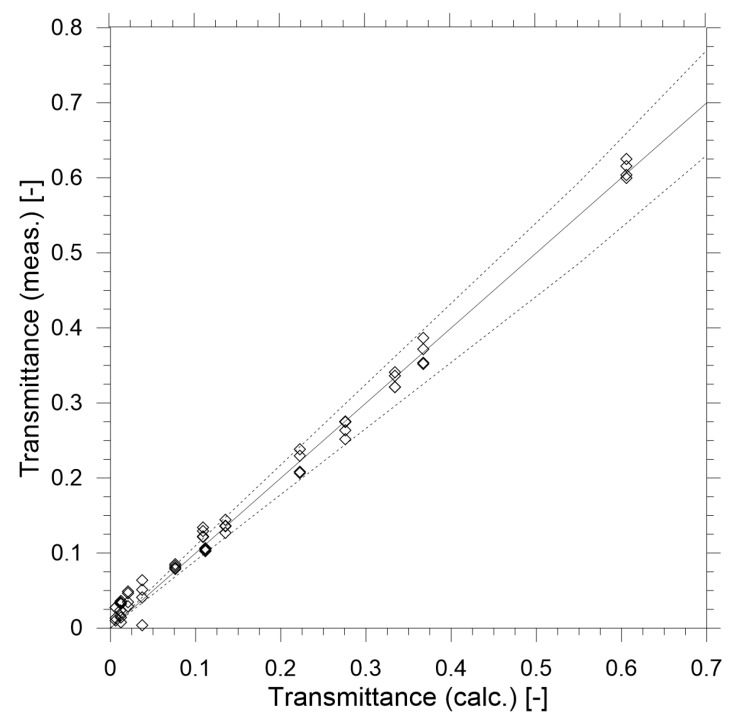
Measured transmittance versus known transmittance value of the calibration filters. Dashed lines represent a 10% difference in measurements.

**Figure 8:**
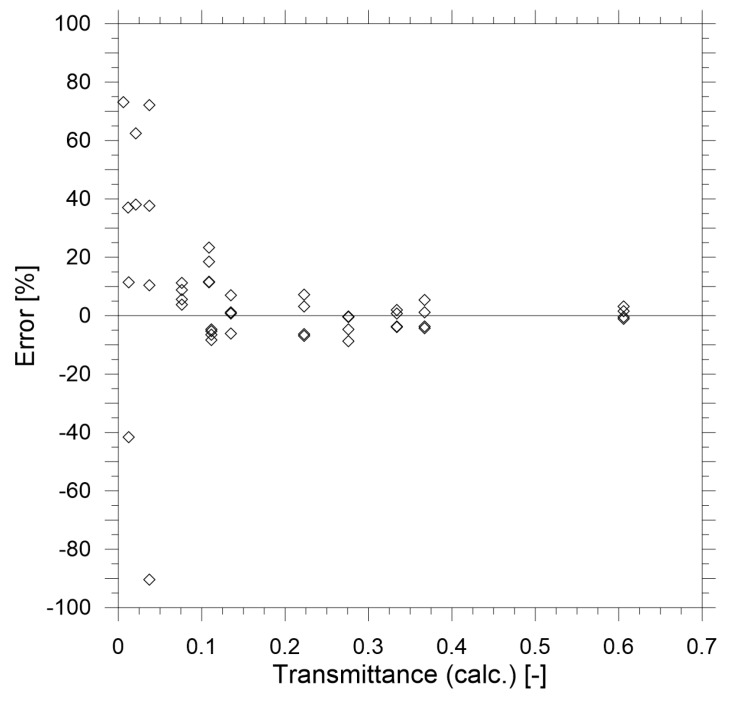
The relative error of transmittance measurements compared to known transmittance value of the calibration filters.

The results of the calibration measurements are shown in Figure 7 and Figure 8. Based on the results of the calibration it may be concluded that in the range of transmittance above 0.25 the performance of the reduced-scale measurement device is within 10% error compared to the theoretical value calculated with Equation (17). This accuracy can be considered satisfactory for the prototype device and it can be improved further by implementing more advanced thermal stabilisation of the device, improved stablization of the electrical supply and use of higher quality components. However, it must be noted that the goal of this study was to create a relatively low-cost device (under $1000, with prismatic mirrors being 75% of the total cost of the prototype).

## 5. Use of Obscuration Measurement in the Reduced-Scale Experiment

### 5.1. Experimental Setup

**Figure 9:**
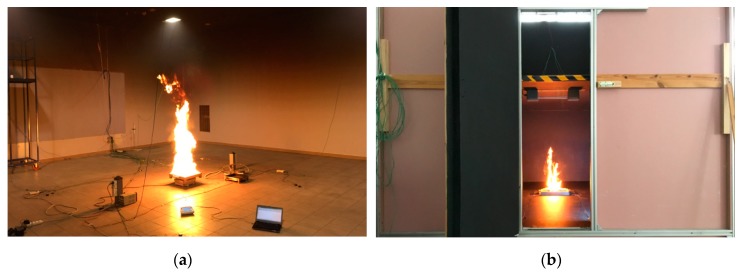
(**a**) Full-scale and (**b**) reduced-scale (1:4) experiments on the free burning of n-heptane.

**Figure 10:**
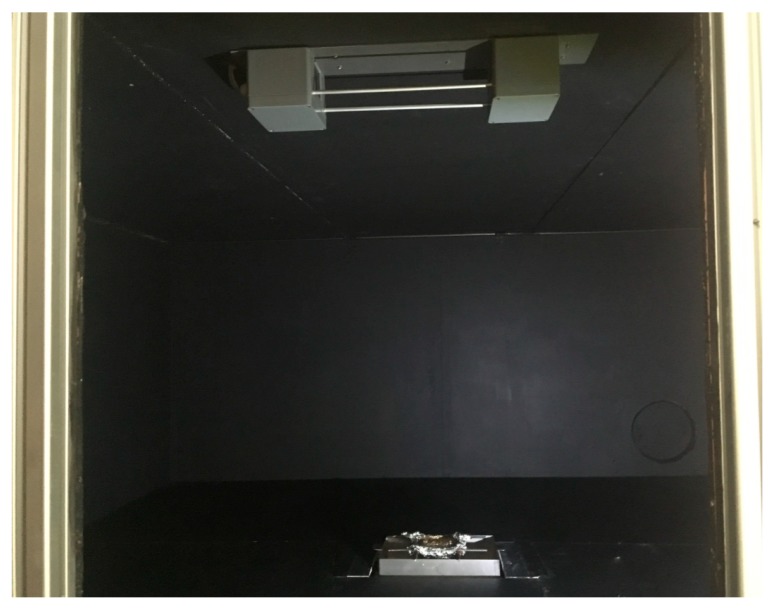
The interior of a reduced-scale (1:4) chamber with visible reduced-scale optical densitometer mounted underneath the ceiling.

To verify the concept of reduced-scale optical densitometer in practice, a series of experiments involving combustion of n-Heptane was performed. The full-scale experiments were performed in the Building Research Institute smoke detector testing chamber (Figure 9a), with dimensions of 9.60 × 9.80 × 4.00 m³. Two experiments each consisting of three repeats were performed. In the first series, a fuel tray with dimensions of 0.33 × 0.33 m² was used (further referred to as series A) and in the second series, a fuel tray with dimensions of 0.50 × 0.50 m² was used (series B). In both full-scale experiments, the fuel was 1 L of n-Heptane. The Heat Release Rate (HRR) was determined through mass loss rate measurements of the fuel tray, with the assumed Heat of Combustion value H_c_ = 44,400 kJ/kg. No ventilation was used in the experiment (the compartment was sealed). The reduced-scale experiment was performed in a scaled-down model of the test chamber (1:4) (Figure 9b and Figure 10), with the dimensions of 2.40 × 2.45 × 1.00 m³. All physical features of the compartment were scaled down accordingly, except the fuel tray. The size of the fuel tray was first determined through geometrical scaling and then refined based on mass-loss measurements of the combustion of n-Heptane so that the similarity of Equations (5) and (9) was explicitly met. The correction to the size of the tray was within 10% of the geometrical size. The similarity of the soot yield factor in both fires was assumed, based on the fact that both fires are considered “small” pool fires, in well-ventilated conditions, with turbulent combustion. However, in research related to bigger fires or more complex fuels, this condition must be rigorously checked.

The summary of the assumptions for the experiment is given in Table 1. Each of the experiments was repeated three times and the conclusions are formed based on the averaged values obtained in the experiments.

**Figure 11:**
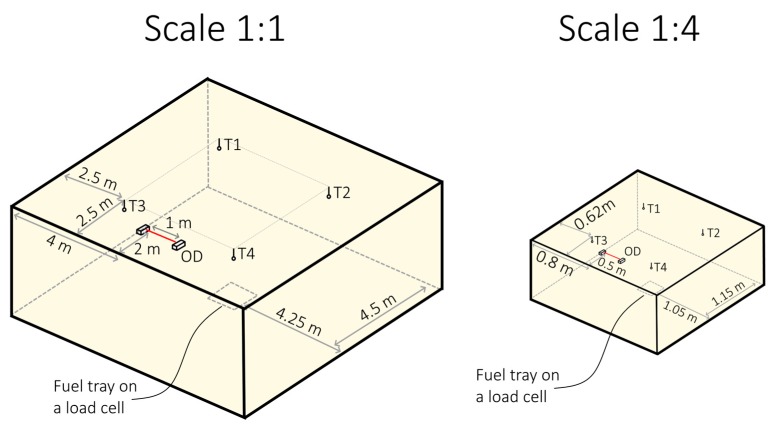
The overview of the test chambers, with the localisation of the measuring equipment (OD—optical densitometer, T1–T4—four thermocouples that were placed symmetrically in four corners of the compartment).

The sketch presenting each of the testing chambers, together with the location of measurement equipment is shown in Figure 11. Each of the testing chambers was equipped with:

- 4 type K 1mm Ni-Cr thermocouples placed in the corners of the compartment, used to measure the average temperature of the smoke layer, extended uncertainty of the measurement is estimated at 0.3 °C;
- load cell with a resolution of 0.01 g was used to measure the mass-loss rate of fuel in the tray in both experiments, with extended uncertainty of 0.02 g;
- optical densitometer: in case of the 1:1 chamber a certified optical densitometer type AML manufactured by Lorenz was used, conforming to EN 14604 [28] and EN 57-7 [29], calibrated with dedicated Lorenz calibration set before the experiment; in case of the 1:4 chamber the reduced-scale optical densitometer as described above. The uncertainty of the optical measurements is difficult to estimate.

### 5.2. Experimental Results

**Figure 12:**
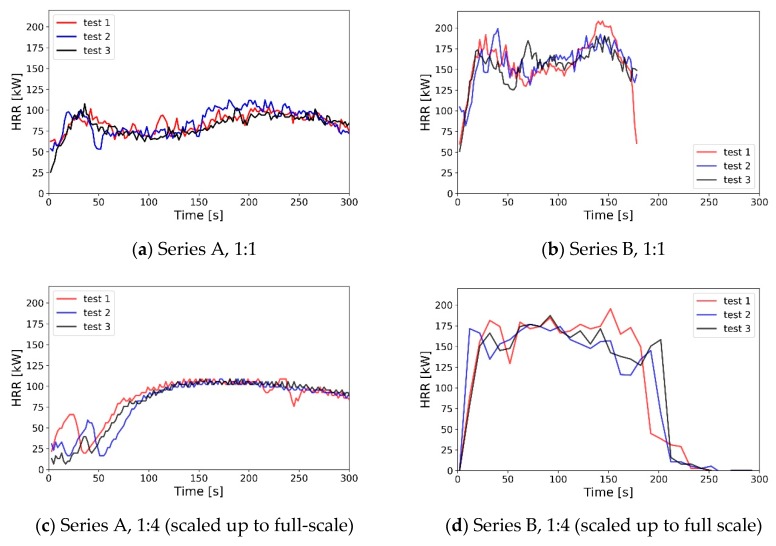
Heat Release Rate (HRR) measurements in the performed experiments—moving averages (5 s averaging time) calculated from mass loss measurements. Values measured in reduced-scale were scaled up based on Equations (5) and (6).

The HRR value for each experiment was plotted based on the moving average value (5 s averaging time) mass loss rate measurements and the assumed Heat of Combustion of the n-Heptane, as shown in Figure 12. The representation of Series A fire in the reduced-scale model in the early part of the experiment was not perfect and differed between the re-runs. However, after the fire stabilised, the obtained average HRR value was roughly similar between full and small scale experiment. The duration of the combustion of the trays was similar between experiments (less than 5% difference after scaling up the time). For experiment series B, both the duration and the transient change of the HRR can be considered similar between the experiments in full- and reduced-scale.

**Figure 13:**
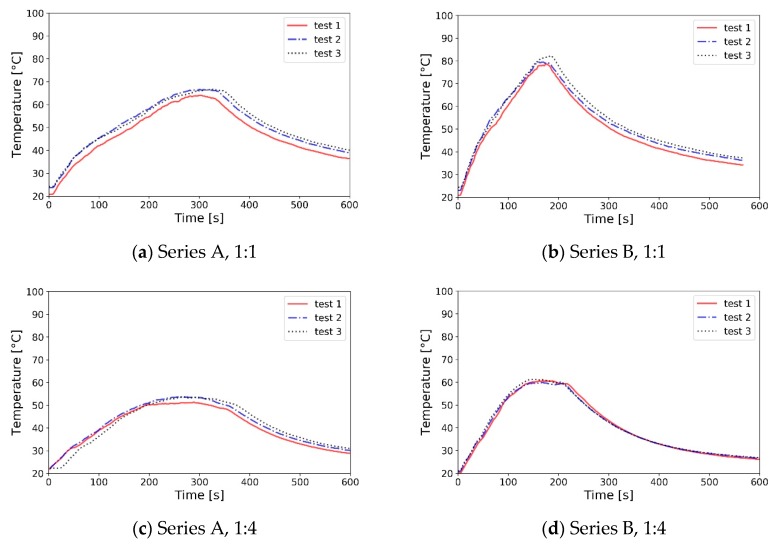
Mean temperature in full- and reduced-scale experiments. Value averaged on four measurements points underneath the ceiling.

The temperature measurements (mean value over four points underneath the ceiling) shows good agreement in terms of the shape of the temperature profile and the peak value timing (Figure 13). In terms of the temperature value, it was noted that the temperatures in the reduced-scale model were up to 6% lower (as measured in K) than in the full-scale experiment. This difference is significant. However, it is in line with the observations from numerical analyses (CFD) and other literature data on Froude-scale modelling [30]. Identification of the cause of the discrepancy will be a subject of further parametric work of the authors. For this research work focused on the development of reduced-scale densitometer, it may be concluded that the conditions of Froude number similarity were sufficiently met between fires in 1:1 and 1:4 scale presented herein.

**Figure 14:**
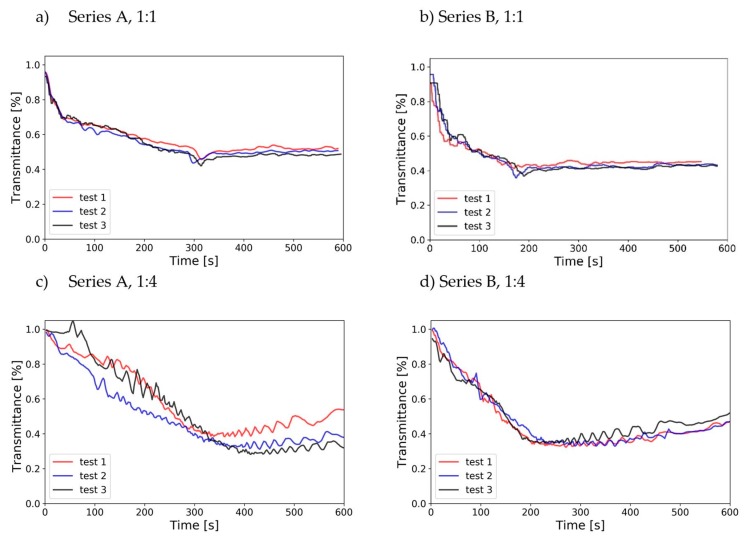
Measurements of transmittance of the hot gas layer in the experiments.

The reduced-scale optical densitometer did capture the change of obscuration density within the reduced-scale model. The results of measurements in 1:1 and 1:4 scales are shown in Figure 14. It can be noticed that the differences in the measurement of the obscuration density between full and reduced-scale are similar to the discrepancies found in the measurement of the Heat Release Rate. However, once the fire stablizes, the measurements agree better, especially after the burnout of the fuel and the stabilisation of the smoke layer. It can be noted that in the full-scale, the change in the first 60 s of the experiment is more rapid than in the reduced-scale, where the rate of change of transmittance is more linear. For the peak obscuration measured, the value obtained in the small scale is slightly lower (approx. 10%) than in the full-scale measurements. It must be noted that a small difference in the measurements may be caused by a difference in peak wavelength of the lasers used in full- and reduced-scale research, which is difficult to quantify. In future research, the full-size chamber will be equipped with a multi-wavelength optical densitometer, that will allow quantifying this error. It was also observed that the measurements in the reduced-scale are noisier than the measurements in the full scale, which may be related to the data acquisition and power supply systems.

## 6. Discussion and Conclusions

This work presents a concept of a reduced-scale optical densitometer that may be used in the measurements of obscuration density of smoke in reduced-scale fire experiments following the Froude number based similarity. The idea of the operation of reduced-scale densitometer is based on the Bougher-Lambert-Beer law and dimensionless groups that describe the scaling of mass, volume, time, and species concentration in the reduced-scale. The physical dimensions of the densitometer are reduced by introducing light-reflecting prisms or mirrors to “wrap” the beam of laser light between the transmitter and receiver into a certain number of shorter beams (corresponding to the scaled-down size of the reference beam in full scale).

This approach allows the performance of obscuration measurements in reduced-scale models, at a moderate cost of the equipment. The total cost of the prototype presented in the paper did not exceed $1000, although the majority of the cost (approximatley $750) was spent on the prismatic mirrors. These mirrors may be replaced with cheaper counterparts (e.g., mirrors for laser applications in CNC), although that may require precision engineering of the mounting unit to maintain correct reflection angles. Furthermore, the precision mirrors may also be used to reflect the laser beam outside of the reduced-scale compartment, so other expensive components such as the laser, filters, or the photodiode are placed outside of the hot smoke zone. As this would introduce additional complexity, this was not performed in the prototype testing. However, the authors do see additional potential benefits coming from improved thermal stabilisation of the device, be it outside of the smoke layer.

The prototype device met the expectations of the authors and did not go through any significant subsequent upgrades between experiments. The transmittance measurement in the reduced-scale was close (up to 10%) to the measurements in the full-scale during combustion. After the layer stabilized, in one of the reduced-scale runs, the discrepancy was approximatley 20% from the averaged measurement in full-scale; for other runs, this was below 10%. A similar difference of results in modelling reduced-scale fires based on Froude similarity was identified for the temperature measurements and will be further researched by the authors in subsequent studies.

The main technical challenges with the use of the reduced-scale densitometer are with the positioning of the laser beam source and the mirrors so that the beam hits precisely the middle of the photodiode. As for the final prototype, IR laser diode was used (980 nm), the location of the beam could not be verified through observation and was controlled by the peak voltage at the photodiode. Based on this experience, for practical reasons, the authors would recommend the use of light in the visible spectrum in future developments (e.g., 633 nm red light). The IR diode was chosen because of two practical reasons: (a) the reference densitometer of the full-scale test chamber works in the IR spectrum and (b) the visible light used for a parallel experiment on emergency lighting could have interfered with the measurements by the reduced-scale densitometer. However, for other developments, these conditions are not relevant and there is no particular justification of the use of IR laser over a visible-spectrum laser.

A significant challenge with the use of this tool may be with the differences observed in the soot production by fires in full and reduced-scale models. These differences come from the differences in the combustion phenomena in various sizes of fires. The user must be well aware of the possible discrepancies this may introduce to the results of the modelling. A simple solution may be to replace the burning source of heat with a plume of hot air with an artificial aerosol introduced to imitate the smoke. In such a setup, the amount of soot could be scaled to the need of the experiment. Furthermore, in research in which all other parameters relevant to the combustion are known and the assessment of the visibility in small scale model is not the primary target, a well-calibrated reduced-scale optical densitometer may be used to estimate the soot yield of a reduced-scale fire.

Finally, besides being a purely scientific tool used in reduced-scale model research, the proposed optical densitometer can play more advanced role of a simulator of a linear optical smoke detector, allowing research in the fields of development of new Artificial Intelligence (AI) based fire detection system networks, such as [31] or in other information-driven fire safety systems such as [32]. This device may enable the use of reduced-scale fire modelling in research in other areas of fire safety science, such as smart disaster management systems employing smart Internet of Things (IoT) based buildings [33], that rely on visibility measurements.

## Figures and Tables

**Table 1:** Overview of the experiments.

Series	Series A		Series B	
Scale	1:1	1:4	1:1	1:4
Heat Release Rate (HRR) [kW]	81.7 kW	2.55 kW	158 kW	4.94 kW
volume of fuel [L]	1.015	0.0158 L	1.015 L	0.0158 L
mass of fuel [g]	0.6943 kg	0.0108 kg	0.6943 kg	0.0108 kg
duration of the fire (real time) [s]	350 s	175 s	181 s	90.5 s
tray size [m]	0.33 × 0.33 m	0.075 × 0.075 m	0.50 × 0.50 m	0.125 × 0.125 m

## Abbreviations

*E*	– energy [J]	Re	– Reynolds dimensionless number
Fr	– Froude dimensionless number	*σ_s_*	– specific mass extinction coefficient [m²/g]
*g_0_*	– the gravitational pull of Earth [N/kg]	*u*	– air flow velocity [m/s]
*I*	– light intensity with smoke [cd]	*t*	– time [s]
*I_0_*	– light intensity without smoke [cd]	*τ*	– transmittance [%]
*l*	– length of the optical path [m]	*x*	– characteristic dimension [m]
*m*	– mass smoke density [kg/m³]	*Y*	– species yield [g/g]
*µ*	– kinematic viscosity [m²/s]	Subscripts	
*n*	– number of optical paths in reduced-scale densitometer [-]	f	– full-scale model
*Q*	– the rate of heat release [kW]	m	– reduced-scale model
*ρ*	– density [kg/m³]	o	– ambient
*R*	– reflectance of a mirror [%]		

## References

[1] Węgrzyński W., Sulik P. (2016). The philosophy of fire safety engineering in the shaping of civil engineering development. Bull. Pol. Acad. Sci. Tech. Sci..

[2] Wald F., Simões da Silva L., Moore D.B., Lennon T., Chladná M., Santiago A., Beneš M., Borges L. (2006). Experimental behaviour of a steel structure under natural fire. Fire Saf. J..

[3] Hidalgo J.P., Cowlard A., Abecassis-Empis C., Maluk C., Majdalani A.H., Kahrmann S., Hilditch R., Krajcovic M., Torero J.L. (2017). An experimental study of full-scale open floor plan enclosure fires. Fire Saf. J..

[4] Ingason H., Li Y.Z., Lönnermark A. (2015). Runehamar tunnel fire tests. Fire Saf. J..

[5] Beji T., Verstockt S., Van De Walle R., Merci B. (2015). Global analysis of multi-compartment full-scale fire tests (’Rabot2012’). Fire Saf. J..

[6] Merci B., Beji T. (2016). Fluid Mechanics Aspects of Fire and Smoke Dynamics in Enclosures.

[7] Quintiere J.G. (1989). Scaling applications in fire research. Fire Saf. J..

[8] Jin T. (1970). Visibility through Fire Smoke (I). Bull. Fire Prev. Soc..

[9] Jin T. (1978). Visibility through fire smoke. J. Fire Flammabl..

[10] Węgrzyński W., Vigne G. (2017). Experimental and numerical evaluation of the influence of the soot yield on the visibility in smoke in CFD analysis. Fire Saf. J..

[11] Mulholland G.W., Liggett W., Koseki H. (1996). The effect of pool diameter on the properties of smoke produced by crude oil fires. Symp. Combust..

[12] Ditch B.D., de Ris J.L., Blanchat T.K., Chaos M., Bill R.G., Dorofeev S.B. (2013). Pool fires—An empirical correlation. Combust. Flame.

[13] Khan M.M., Tewarson A., Chaos M. (2016). Combustion Characteristics of Materials and Generation of Fire Products. SFPE Handbook of Fire Protection Engineering.

[14] Thomas P.H., Hinkley P.L., Theobald C.R., Simms D.L. (1963). Investigations Into the Flow of Hot Gases in Roof Venting.

[15] Prahl J., Emmons H.W. (1975). Fire induced flow through an opening. Combust. Flame.

[16] Quintiere J.G. (2006). Fundamentals of Fire Phenomena.

[17] Williams F.A. (1969). Scalling Mass Fires. Fire Research Abstracts and Reviews.

[18] Jones L.A. (1923). An instrument (densitometer) for the measurement of high photographic densities. J. Franklin Inst..

[19] Baker E.A. (1924). A convenient photo-electric photometer and densitometer. J. Sci. Instrum..

[20] Jin T. (1971). Visibility through Fire Smoke (II). Bull. Fire Prev. Soc..

[21] Mulholland G.W., Croarkin C. (2000). Specific extinction coefficient of flame generated smoke. Fire Mater..

[22] Choi M.Y., Hamins A., Mulholland G.W., Kashiwagi T. (1994). Simultaneous optical measurement of soot volume fraction and temperature in premixed flames. Combust. Flame.

[23] Garcés H., Fuentes A., Reszka P., Carvajal G., Garcés H.O., Fuentes A., Reszka P., Carvajal G. (2018). Analysis of Soot Propensity in Combustion Processes Using Optical Sensors and Video Magnification. Sensors.

[24] Tang R., Zhang T., Wei X., Zhou Z., Tang R., Zhang T., Wei X., Zhou Z. (2017). An Efficient Numerical Approach for Field Infrared Smoke Transmittance Based on Grayscale Images. Appl. Sci..

[25] Widmann J.F. (2003). Evaluation of the planck mean absorption coefficients for radiation transport through smoke. Combust. Sci. Technol..

[26] Widmann J.F., Duchez J., Yang J.C., Conny J.M., Mulholland G.W. (2005). Measurement of the optical extinction coefficient of combustion-generated aerosol. J. Aerosol Sci..

[27] McGrattan K., Hostikka S., McDermott R., Floyd J., Weinschenk C., Overholt K. (2017). Fire Dynamics Simulator User’s Guide.

[28] CEN EN 14604:2005+AC:2008 Smoke Alarm Devices. https://www.sis.se/api/document/preview/67895/.

[29] CEN EN 54-7:2018 Fire Detection and Fire Alarm Systems - Part 7: Smoke Detectors - Point Smoke Detectors That Operate Using Scattered Light, Transmitted Light or Ionization. https://standards.globalspec.com/std/13061688/EN%2054-7.

[30] Barsim M.M., Bassily M.A., El-Batsh H.M., Rihan Y.A., Sherif M.M. (2019). Froude scaling modeling in an Atrium Fire equipped with natural and transient forced ventilation. Int. J. Vent..

[31] Park J.H., Lee S., Yun S., Kim H., Kim W.T., Park J.H., Lee S., Yun S., Kim H., Kim W.T. (2019). Dependable Fire Detection System with Multifunctional Artificial Intelligence Framework. Sensors.

[32] Li Y., Wang A., Yi X., Li Y., Wang A., Yi X. (2019). Fire Control System Operation Status Assessment Based on Information Fusion: Case Study. Sensors.

[33] Park S., Park S., Park L., Park S., Lee S., Lee T., Lee S., Jang H., Kim S., Chang H. (2018). Design and Implementation of a Smart IoT Based Building and Town Disaster Management System in Smart City Infrastructure. Appl. Sci..

